# P-2043. Improving Access and Adherence to Antiretroviral Therapy Through Coordinated Outreach and Care Linkage Model: A Collaborative Program Evaluation

**DOI:** 10.1093/ofid/ofaf695.2207

**Published:** 2026-01-11

**Authors:** Patrick Ryscavage, Natalie Spicyn, Jonathan Darer, Michael Bogart

**Affiliations:** University of Maryland, Baltimore, Baltimore, MD; University of Maryland, Baltimore, Maryland; Health Analytics, Baltimore, Maryland; Gilead Sciences, Inc., Foster City, CA, United States, Foster City, California

## Abstract

**Background:**

People with HIV (PWH) who receive ongoing outpatient HIV care have improved viral suppression, higher CD4 counts, and reduced mortality (Ulett 2009). Hospitalization is a vital opportunity to assess participation in HIV care and re-engage out-of-care PWH. The University of Maryland, Baltimore (UMB) created a multi-disciplinary care linkage program (UCLP) that engages hospitalized PWH and follows them post-discharge to facilitate participation in HIV care, including attendance at outpatient visits and collaboration outside the UMB system until the individual is successfully linked. This study evaluated the impact of UCLP on linkage to care.

**Methods:**

This study was a retrospective comparative cohort analysis of PWH for whom UCLP was part of their discharge plan (Participants) compared to those for whom their discharge plan did not include UCLP (Controls). Enrollment criteria included either a new diagnosis of HIV or pre-existing diagnosis and documented out-of-care status during the 12 months prior to hospitalization. The primary outcome was successful linkage to care as defined by two visits with an HIV clinic at least 60 days apart.

**Results:**

Participants (N=79) and controls (N=62) had an average age of 50.2 years and 49.0 years, respectively. Individuals were predominantly Black/African American (90% vs 84%; n.s.), male (57% vs 66%; n.s.), and on Medicaid (70% vs 81%; n.s.). IV drug use during the past 12 months was present in 27% of participants and 36% of controls (n.s.). Both participants and controls experienced comorbid depression (30% vs 24%; n.s.), anxiety (13% vs 8%; n.s.), and bipolar disorder (11% vs 20%; n.s.). On admission to the hospital, opportunistic infections were present in 20% of participants and 15% of controls. New diagnoses of HIV were less common among participants (14%) compared to controls (31%). Successful linkage to care was statistically significantly greater among participants (63%) compared to controls (11%; p < .001). ART was prescribed at discharge in 72% participants and 58% of controls (n.s.).

**Conclusion:**

The UCLP program was associated with a significantly higher rate of linkage to care in hospitalized PWHs transitioning to outpatient care. Inpatient linkage to care programs represent a high-yield opportunity to support continuity of care for PWH.

**Disclosures:**

Patrick Ryscavage, MD, Gilead Sciences, Inc.: Grant/Research Support Jonathan Darer, MD, MPH, Acadia: Grant/Research Support|Boehringer Ingelheim: Grant/Research Support|Caresyntax: Grant/Research Support|Gilead Sciences, Inc.: Grant/Research Support|GSK: Grant/Research Support|iota: Grant/Research Support|Johnson & Johnson: Grant/Research Support|Kenvue: Grant/Research Support|Mentavi: Grant/Research Support|Novo Nordisk: Grant/Research Support|Osteal: Grant/Research Support|UCB: Grant/Research Support Michael Bogart, n/a, Gilead Sciences, Inc.: Employee|Gilead Sciences, Inc.: Stocks/Bonds (Public Company)

